# Motivation in psychocardiological rehabilitation

**DOI:** 10.3389/fpsyg.2013.00827

**Published:** 2013-11-06

**Authors:** Giada Pietrabissa, Gian Mauro Manzoni, Gianluca Castelnuovo

**Affiliations:** ^1^Psychology Research Laboratory, Istituto Auxologico Italiano IRCCS, Ospedale San GiuseppeVerbania, Italy; ^2^Department of Psychology, Catholic University of MilanMilan, Italy

**Keywords:** motivation, cardiological rehabilitation, stroke rehabilitation, psychocardiology, cardiac psichology

In this book a group of independed-minded academics has orchestrated a multi-authored text that represents an update of knowledge concerning how motivation influences Cardiovascular (CV) response.

Discussing diverse motivational approaches that are representative of works carried out in several areas of psychology, health implications of the linkages between motivational variables and CV response have been investigated.

The volume is divided into two major parts: Part I includes chapters that concern mechanisms of motivational influence on CV response, while in the Part II motivational paths to carry out with the aim of investigate CV response in different life circumstances have been considered.

In more detail, 4 subsections organize Part I through 10 chapters, and 3 subsections constitute Part II by means of 9 chapters, each of those preceded by a summary of the key points.

The opening chapters discuss the importance of modern neuroimaging techniques in allowing occasions to investigate how the interplay between CV status, cognitive-emotional functioning, and environmental demands are realized within the central and peripheral nervous systems.

The relationship between emotional arousal and CV response has been noteworthy argued and enriched by results from recent research findings in various disciplines (Pietrabissa et al., 2012; Compare et al., 2013a). These studies demonstrate stress, motivational intensity, individual personality, self-regulation and mood states being in charge of health damaging CV adjustments, such as heart rate, blood pressure, and heart contraction force (Compare et al., 2011a, 2012, 2013b).

Part II mainly contains evidences that CV activity is an indicator or even cause for CV diseases development, also including explanations about the role covered by gender, psychological processes, and social interactions in influencing health outcomes via relevant biological pathway. In addition, a set of considerations that may guide future research on this subject are discussed.

Due to the various contributions from different authors of different countries, both style of writing and chapters arrangement widely varies.

Some issues are effectively revisited on several occasions in the volume, constituting minor imperfections in an otherwise excellent, comprehensive, and up-to-date overview of mechanisms and applications related to CV disease. In fact, this book, edited by two psychologists and written by an International group of specialists in different fields of medicine, successfully covers the subject.

Because of diseases generally display a multifactorial aetiology and symptoms require to be assumed as manifestations of a complex interplay of several factors, the bio-psycho-social approach of illness theorized by Engel (1977) remains in the background. Health and illness are not seen as opposite concepts or distinct entities but extremes of a continuum on which individuals constantly flux depending on the quality of their daily-life experiences, influenced by biological (e.g., genetics, age), psychological (e.g., attitude, stress) (Compare et al., 2011b, 2013c), and social (e.g., interpersonal relationships, socio-economic status) factors (Castelnuovo, 2010; Compare et al., 2013c). Due to this complexity, as well as stated the crucial role of both personal dispositions and intentions in modifying personal attitudes and behaviors, the relevance of setting realistic goal, implementing patient-centered treatments and developing motivational enhancement techniques, is highlighted.

Overall, this book represents a well-collated collection of research outcomes, successfully demonstrating the importance of both psychological factors and motivation in the treatment of patients suffering from CV diseases (Manzoni et al., 2011a). In fact, as extensively discussed through the pages of this volume, the individual's level of initial motivation, reflecting the maximum energy spent by a person for goals attainment, represent one the main predictor of success in preventing and managing CV risk factors (Manzoni et al., 2011b) also by modern telecare systems (Villani et al., 2007). However, the text is so densely written than chapters seem to be resources for references rather than flowing text. Despite recent technological changes have made information readily available on the Internet, because of potentially appealing scholars and practitioners from a wide range of areas, together with its acceptable price, this book still constitute a significant source of outcomes and data.
